# Effect of probiotic *Bifidobacterium bifidum* G9-1 on the relationship between gut microbiota profile and stress sensitivity in maternally separated rats

**DOI:** 10.1038/s41598-018-30943-3

**Published:** 2018-08-17

**Authors:** Hirokazu Fukui, Tadayuki Oshima, Yoshiki Tanaka, Yosuke Oikawa, Yutaka Makizaki, Hiroshi Ohno, Toshihiko Tomita, Jiro Watari, Hiroto Miwa

**Affiliations:** 1 Division of Gastroenterology, Department of Internal Medicine, Hyogo College of Medicine, Nishinomiya, Japan; 2 R&D Center, Biofermin Pharmaceutical Co., Ltd, Kobe, Japan; 3 Medical Science Department, Biofermin Pharmaceutical Co., Ltd, Kobe, Japan

**Keywords:** Physiology, Irritable bowel syndrome

## Abstract

Although gut microbiota and early life events are likely involved in the development of irritable bowel syndrome (IBS), it remains unclear how these factors interact in the pathophysiology of IBS. In the present study, using rats subjected to maternal separation (MS) as a model of IBS, we investigated interrelationships among gut microbiota, stress susceptibility and intestinal permeability, and examined the effect of the probiotic *Bifidobacterium bifidum* G9-1 (BBG9-1) on those interrelationships. When compared with the controls at postnatal day 20, MS rats showed hypercorticosteronemia, enhanced intestinal permeability and changes in gut microbiota structure. All of these changes in MS rats were prevented by treatment with BBG9-1. Although the gut microbiota profile and basal serum corticosterone level did not differ between MS and control rats at postnatal day 56, MS rats showed hypersensitivity to restraint stress in terms of serum corticosterone level and fecal frequency. However, such hypersensitivity was not observed in MS rats treated with BBG9-1. These findings suggest that MS initiates the link between gut microbiota alteration and hypersensitivity to stress and that the triggering of this process can be prevented by the treatment with the probiotic BBG9-1.

## Introduction

Irritable bowel syndrome (IBS) is classified as a functional gastrointestinal disorder (FGID) characterized by symptoms such as abdominal pain or discomfort, bloating and stool irregularities, without any apparent evidence of structural or organic lesions^1^. The pathogenesis of IBS appears to be multifactorial, including psychological factors, neuroimmune interactions, gut microbiota, the neuroendocrine system and dietary factors^2^. Among them, much attention has recently been paid to the gut microbiota in IBS patients because its alteration importantly affects homeostasis of the mucosal barrier, gut sensorimotor function and the brain-gut axis^3–5^. In this context, probiotics have been applied as therapeutic drugs to normalize microbiota imbalance, although there is still no evidence that they have significant therapeutic effectiveness in patients with IBS^6–8^. Moreover, it remains unclear which bacterial strains are responsible for IBS development, although the gut microbiota profile is likely to differ between healthy individuals and IBS patients^2,3^.

Accumulating evidences has revealed that probiotics are useful for the treatment of patients with IBS^9,10^, suggesting a pivotal role for gut microbiota in the pathophysiology of IBS. Among various probiotics, *Bifidobacterium* appears to be a promising candidate for treatment of IBS although it is difficult to compare the efficacy of probiotics for IBS due to the variety of experimental designs employed or the clinical factors evaluated^9,10^. In this context, it seems valuable to examine the effect of *Bifidobacterium bifidum* G9-1 (BBG9-1) for the treatment of IBS. BBG9-1 isolated from a healthy individual has been used as an intestinal medicine for several decades. Furthermore, we have recently reported that BBG9-1 has a mucosal protective effect against virus-induced enteritis^11^ and can attenuates anti-cancer drug-induced inflammation by helping to ameliorate dysbiosis^12^. Accordingly, we were motivated to examine the effect of BBG9-1 in an animal model of IBS.

To clarify the effects of probiotics on the pathophysiology of IBS, not only clinical but also experimental studies are needed. Rodents subjected to neonatal maternal separation (MS) constitute a well-established model that exhibits features similar to those of IBS in humans, such as visceral hyperalgesia and enhanced colonic motility in response to acute stress^13–15^. As seen in this animal model, it is known that childhood abuse is indeed closely associated with the development of functional gastrointestinal diseases such as functional dyspepsia^16^ and IBS^17^. This suggests that traumatic events in early life have a critical role in initiating long-lasting gut dysfunction that persists throughout life. However, it has been unclear how such traumatic events play a role in the development of IBS, and how IBS might be therapeutically targeted. In the present study using the MS rat model of IBS, we focused on the interrelationships existing among gut microbiota, stress susceptibility and gut pathophysiology, and examined the effect of the probiotic BBG9-1 in this context.

## Methods

### Animals

Pregnant Sprague-Dawley rats were obtained from Charles River Laboratories Japan (Yokohama, Japan) on gestational day 15. They were housed under a 12:12-h light-dark cycle (lights on at 7 am) with free access to food (CE-2; Clea Japan, Tokyo, Japan) and water. Temperature was maintained at 22 ± 3 °C and humidity at 55 ± 5%. All experimental procedures were approved by the Experimental Animal Care and Use Committee of Biofermin Pharmaceutical Co., Ltd.

### Probiotics

BBG9-1 (*Bifidobacterium bifidum* G9-1; Biofermin R&D Center, Kobe, Japan)^11^ was obtained from the Culture Collection of Biofermin Pharmaceutical Co., Ltd. and cultured at 37 °C for 18 h in GAM Broth (Nissui Pharmaceutical. Co., Ltd., Tokyo, Japan) supplemented with 0.7% glucose and 0.1% Tween 80. The bacterial cells were recovered by centrifugation at 3000 × *g* for 15 min.

### Maternal separation and experimental design

**Figure 1:**
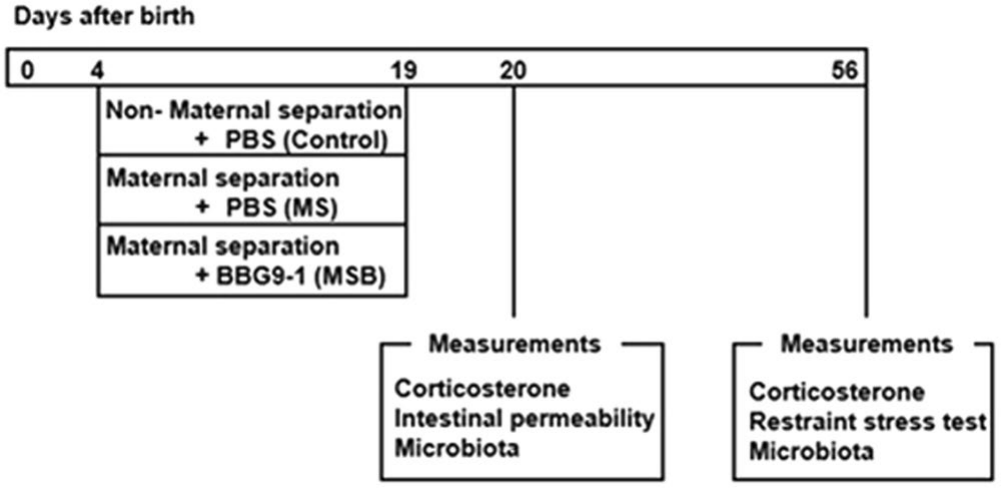
Experimental design of maternal separation and restraint stress test.

MS was performed according to the protocol described by Gareau *et al*.^18^. In brief, dams and their litters (culled to 10 pups comprising equal numbers of males/females) were randomly assigned to the MS protocol or to the control non-separation protocol. MS pups were individually separated from their dams for 3 h (9:00–12:00) per day from days 4 to 19 of life, whereas control pups were left in their home cages with their dams. Pups subjected to MS were removed from their home cage and transferred to individual cages lined with chip bedding and maintained in a standard room at 37 ± 0.5 °C with heating pads. On the other hand, the dam was moved to a separate room. From days 4 to 19 of life, a proportion of the MS rats were orally gavaged with 50 μl of PBS containing 1 × 10^8^ CFU BBG9-1 once daily immediately after removal from their dam, whereas other rats were given the same amount of PBS alone as a placebo. Thus, the following experimental groups were created: Control group subjected to neither MS nor BBG9-1 treatment; MS group subjected to MS without BBG9-1 treatment; MSB group subjected to MS with BBG9-1 treatment. Thereafter, male rats were subjected to analysis of mucosal integrity and a restraint stress test at postnatal days 20 and 56 days, respectively (Fig. 1). Final MS was carried out at postnatal day 19. The rats were weaned at postnatal day 20, then housed at two rats per cage. At postnatal day 20 (between 1 and 3 pm), 24 h after the end of final MS, a blood sample was collected from the inferior vena cava of each rat under isoflurane anesthesia.

### Extraction of DNA from fecal samples

Extraction of bacterial DNA for sequencing was performed as described previously^19^. Fecal samples (20 mg) were collected from rats immediately after defecation at postnatal days 20 and 56, washed three times by suspending them in 1.0 ml of PBS, and centrifuged (14,000 × *g*) to remove possible PCR inhibitors. The fecal pellets were resuspended in a solution containing 450 μl of extraction buffer (100 mM Tris-HCl, 40 mM EDTA; pH 9.0) and 50 μl of 10% sodium dodecyl sulfate. Three hundred milligrams of glass beads (diameter, 0.1 mm) and 500 μl of buffer-saturated phenol were added to the suspension, and the mixture was vortexed vigorously. After centrifugation at 14,000 × *g* for 5 min, 400 μl of the supernatant was collected. Subsequently, phenol-chloroform extractions were performed, and 250 μl of the supernatant was subjected to isopropanol precipitation. Finally, the DNA was suspended in 1 ml of Tris-EDTA buffer.

### Illumina library generation and DNA sequencing

Analysis of the 16S rDNA of the microbial community presents in feces was performed in accordance with a method described previously^20^ with minor modifications. In brief, the V3-V4 region of 16S rDNA was amplified using a forward primer (5′-TCGTCGGCAGCGTCAGATGTGTATAAGAGACAGCCTACGGGNGGCWGCAG-3′) and a reverse primer (5′-GTCTCGTGGGCTCGGAGATGTGTATAAGAGACAGGACTAC HVGGGTATCTAATCC-3′), which were ligated with overhang Illumina adapter consensus sequences. The PCR was performed using the following program: 95 °C for 3 min, followed by 25 cycles consisting of 95 °C for 30 s, 55 °C for 30 s and 72 °C for 30 s. After 25 cycles, the reaction was completed with a final extension of 5 min at 72 °C on a Veriti thermal cycler (Thermo Fisher Scientific, Waltham, MA, USA). The amplicon was purified using AMPure XP magnetic beads (Beckman Coulter, Brea CA, USA). The Illumina Nextera XT Index kit (Illumina) with dual 8-base indices was used to allow for multiplexing. To incorporate two unique indices to the 16S amplicons, PCR reactions were performed. Cycling conditions consisted of 95 °C for 3 min, followed by eight cycles of 95 °C for 30 s, 55 °C for 30 s and 72 °C for 30 s, followed by a final extension cycle of 72 °C for 5 min. After purification by AMPure XP beads, the purified barcoded library was quantified fluorometrically using a QuantiT PicoGreen ds DNA Assay Kit (Invitrogen, Paisley, UK). Libraries were then diluted to 4 nM using 10 mM Tris-HCl (pH 8.0), followed by pooling of the same volume for multiplex sequencing. The multiplexed library pool (10 pM) was spiked with 40% PhiX control DNA (10 pM) to improve base calling during sequencing. Sequencing was conducted using a 2 × 250-bp paired-end run on a MiSeq platform with MiSeq Reagent Kit v2 chemistry (Illumina).

### DNA sequence analysis

Demultiplexing and removal of indices were performed using the MiSeq Reporter software (Illumina). Subsequently, sequence files were exported from the MiSeq Reporter software as a further step. Filtering out of low-quality sequences, removal of chimera sequences, construction of operational taxonomic units (OTUs), and taxonomy assignment was conducted using the Quantitative Insights Into Microbial Ecology (QIIME) pipeline (http://qiime.org/)^21^. In brief, 30000 raw reads were randomly obtained from the sequence files for each sample and merged by fastq-join with the default setting. Consequently, sequence reads with an average quality value of <25 were removed, and then chimera-checked. Five thousand high-quality sequence reads were randomly obtained for each sample, and OTUs for total high-quality reads were constructed by clustering with a 97% identity threshold. The representative reads of each OTU were then assigned to the 16S rRNA gene database by using UCLUST with ≥97% identity. Comparison of each taxon in gut microbiota was conducted at genus level. Beta diversity was estimated by computing weighted UniFrac distance between samples, a phylogenic tree-based metric^22^. To compare the differences in the overall bacterial gut microbiota structure, principal co-ordinates analysis was applied to reduce the dimensionality of the resulting distance matrix. The Shannon index was calculated to investigate the alpha diversity of microbiota in the samples.

### Mucosal integrity

Rats were orally administered fluorescein isothiocyanate (FITC) dextran (average molecular weight, 4000; Sigma-Aldrich, St.Louis, MO, USA) at a dose of 750 mg/kg in PBS^23^. After 1 hour, blood was obtained from the inferior vena cava using heparin-coated capillaries. Samples were centrifuged (2000 × *g* for 10 min), and the plasma concentration of FITC was measured with a microplate reader (excitation, 485 nm and emission, 525 nm; infinite M200PRO, TECAN, Grödig, Austria).

### Ussing chamber studies

Segments of the distal colon were cut along the mesenteric border and mounted in Ussing chambers (Physiologic Instruments, San Diego, CA, USA), exposing a tissue area of 0.3 cm^2^ to circulating oxygenated Krebs buffer (115 mM NaCl, 1.25 mM CaCl_2_, 1.2 mM MgCl_2_, 2.0 mM KH_2_PO_4_, 25 mM NaHCO_3_) at 37 °C. Mannitol (10 mM) and glucose (10 mM) were additionally included in the Krebs buffer for the mucosal and serosal sides, respectively. Thereafter, the short-circuit current (Isc) was recorded as described previously^14^. Briefly, Isc values were obtained at equilibrium, ~20 min after the tissues had been mounted, and expressed as μA/cm^2^.

### Restraint stress and serum corticosterone

Acute restraint stress was carried out as reported previously^24^. The stress cage (stainless steel, W70 × L55 × H230 mm, Natsume Seisakusho Co., Ltd., Tokyo, Japan) used in this study was adjustable to the body of the rats body with an inner fixture allowing access to the tail for blood sampling, and each rat was placed inside the stress cage for 1 hour. Blood samples were obtained from the tail vein of conscious rats before and after (30 and 60 min) the stress. Serum was isolated by centrifugation (2000 × *g* for 20 min) and stored at −30 °C until ELISA assay. In addition, the number of fecal pellets egested was counted during this stress test. The serum corticosterone level was measured using a Corticosterone ELISA kit (Enzo Life Sciences, Farmingdale, NY, USA).

### Statistical analysis

All statistical analyses were conducted with the R statistical software version 3.1.3.^25^. Data are shown as means ± SE. Statistical significance was set at *p* < 0.05. Normality tests were initially performed for analysis of data sets. When a data set was not modeled by a normal distribution, statistical significance was determined by the Steel-Dwass test. When it was well modeled by a normal distribution, its homoscedasticity was analyzed by Bartlett’s test. If a data set was homoscedastic, statistical significance was determined using the Tukey-Kramer or Dunnett test. If heteroscedasticity was evident, statistical significance was determined using the Steel-Dwass test. In the analyses of gut microbiota, statistical significance was determined by Welch’s t test with Benjamini-Hochberg correlation. The statistical analyses used for each experiment are described in the respective figure legends.

### Ethics approval and consent to participate

The animal experiments were carried out with the approval of the Experimental Animal Care and Use Committee of Biofermin Pharmaceutical Co., Ltd.

## Results

### Effect of BBG9-1 on body growth, serum corticosterone and gut microbiota profile in MS rats

**Figure 2:**
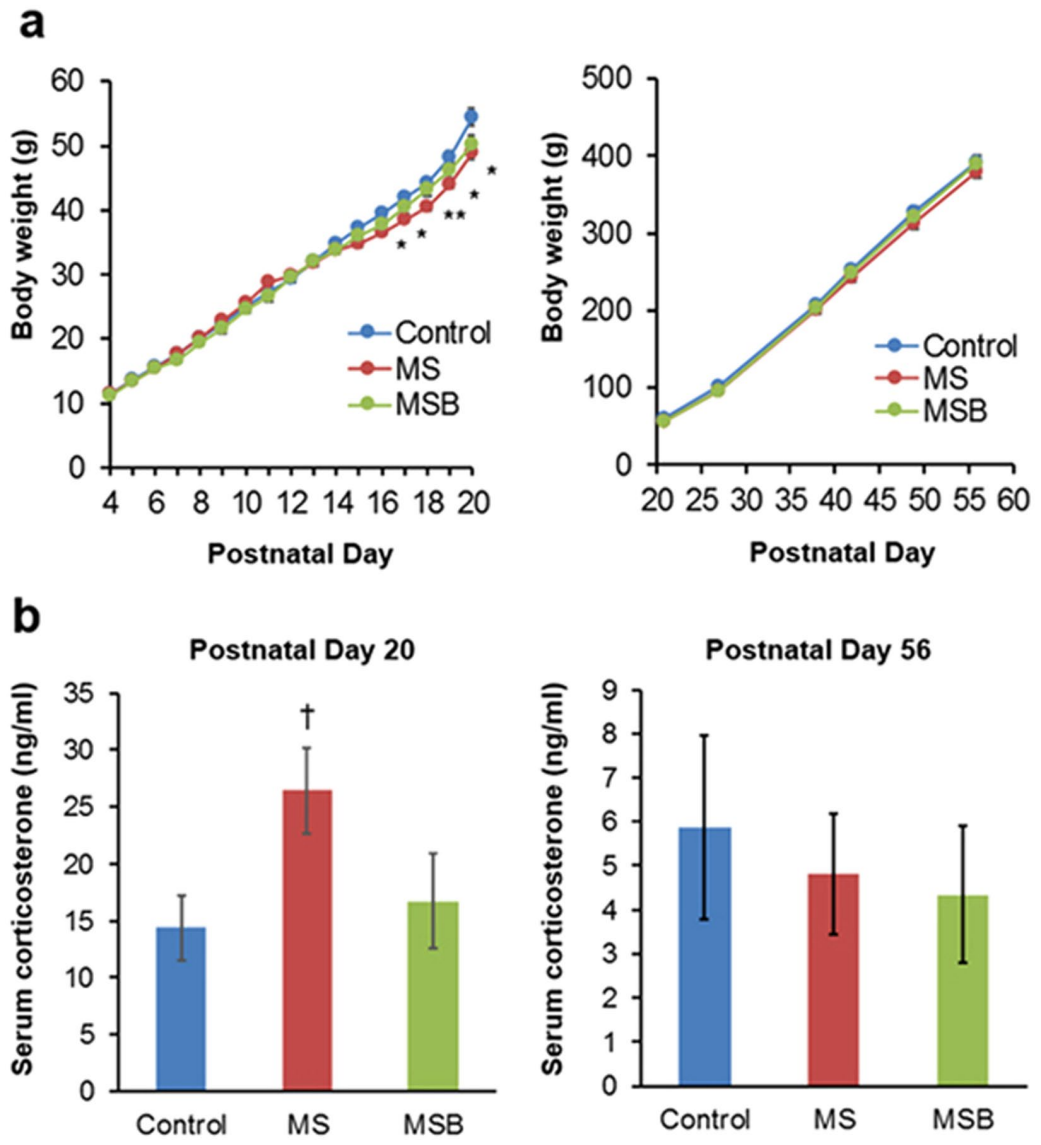
Effect of BBG9-1 on body growth and serum corticosterone level in MS rats. (**a**) Change in body weight (n = 8 rats per group). (**b**) Serum corticosterone level (n = 8 rats per group) at postnatal days 20 and 56. MS, maternal separation; MSB, MS with BBG9-1 treatment. Results are expressed as the mean ± SE. Significantly smaller than in the control at the same time point: *P < 0.05, **P < 0.01 (Steel-Dwass test). Significantly higher than the control: ^†^*P* < 0.05 (Dunnett test).

Serial changes in body weight are shown in Fig. 2a. The body weight of rats in the MS group was significantly less than that of controls from postnatal days 16 to 20 (Day16~19; F(2) > 3.4668, *p* < 0.05, Tukey-Kramer post hoc [Ctrl vs MS] *p* < 0.05, Day20; Kruskal-Wallis test *p* < 0.05, Steel-Dwass post hoc [Ctrl vs MS] *p* < 0.05), suggesting that chronic stress produced by MS had an inhibitory effect on body growth in infant rats. On the other hand, although BBG9-1 treatment did not completely ameliorate the MS-induced inhibition of body growth, no significant difference in body weight was evident between control and MSB rats, suggesting that BBG9-1 treatment had at least a partly preventive effect on MS-induced inhibition of body growth. Further follow-up of body weight in the three rat groups revealed no significant differences beyond postnatal day 21, suggesting that the inhibitory effect of MS on body growth was transparent and reversible.

To assess the activity of the hypothalamus-pituitary-adrenal axis, we examined the serum corticosterone level (Fig. 2b). The level of corticosterone in the MS group was significantly higher than that in the control group at postnatal day 20 (Dunnett test [Ctrl vs MS] *p* < 0.05). This MS-induced increase in the corticosterone level was attenuated by treatment with BBG9-1 at this time point. At postnatal day 56, there were no significant differences in the basal corticosterone level among the three groups (Kruskal-Wallis test *p* = 0.728) (Fig. 2b) although the levels were lower than those at postnatal day 20. This discrepancy of the basal serum corticosterone level may have been due, at least in part, to the age of the rats or the methods used for blood sampling.

**Figure 3:**
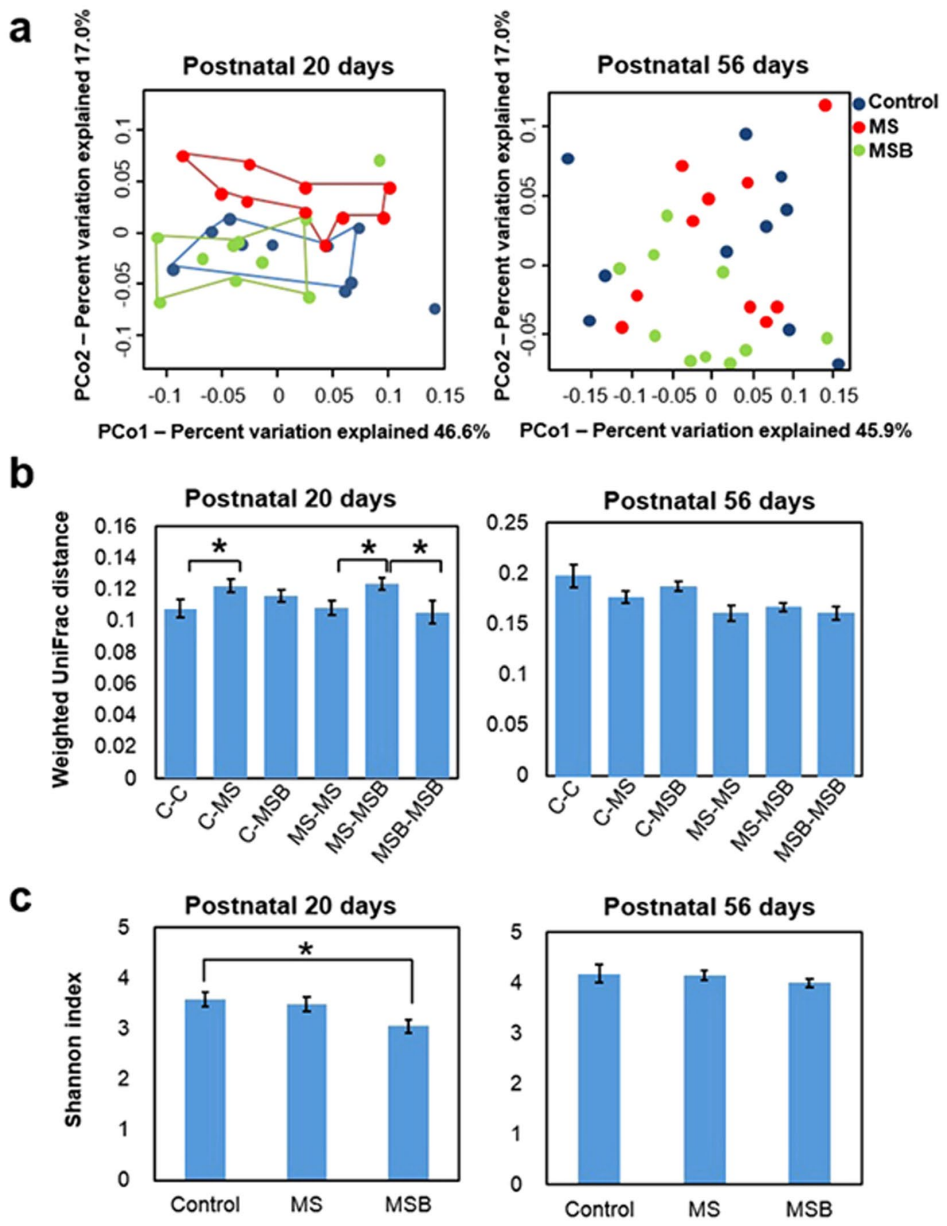
Effect of BBG9-1 on the structure of gut microbiota in MS rats. (**a**) Weighted UniFrac Principal Coordinate analyses (PCoA) showing the clustered communities of intestinal microbiota in the experimental rats. PCo1 and PCo2 explained the indicating percentage of variation on the x-axes and y-axes, respectively. (**b**) Weighted UniFrac distance analyses between the control, maternal separation (MS), and MS with BBG9-1 treatment (MSB) groups (n = 10 in each group). Each column represents the mean weighted UniFrac distance within the control (C-C), MS (MS-MS) or MSB group (MSB-MSB) and between the control and MS (C-MS), control and MSB (C-MSB), or MS and MSB (MS-MSB) group. (**c**) Alpha-diversity of gut microbiota. Shannon index calculated from the observed OTU numbers of intestinal microbiota samples from postnatal day 20 and 56. Results are expressed as the mean ± SE (n = 10 in each group). Asterisks indicate statistical significance determined by Welch’s t test with Benjamini-Hochberg correlation or Tukey-Kramer test (*p < 0.05).

We then investigated the effect of BBG9-1 on gut microbiota structure in MS rats. Weighted UniFrac-based principal coordinate analysis (PCoA) revealed differential clustering of the gut microbiota structures between MS and control rats at postnatal day 20 (Fig. 3a), indicating that MS induced an alteration in the gut microbiota structure. On the other hand, the structure of the gut microbiota in BBG9-1-treated rats was similar to that in normal controls, suggesting that BBG9-1 treatment exerted a normalization effect on the gut microbiota structure after alteration by MS. Indeed, UniFrac distance analyses showed that the distance between control and MS rats was significantly larger than the distance within normal rats (Welch’s t-test with Benjamini-Hochberg correlation [Ctrl-Ctrl vs Ctrl-MS] *p* < 0.05), whereas the distance between control and BBG9-1-treated rats was not different from the distance within normal rats ([Ctrl-Ctrl vs Ctrl-MSB] *p* = 0.298) (Fig. 3b). The distance between MS and BBG9-1-treated rats was significantly larger than not only the distance within MS rats (Welch’s t-test with Benjamini-Hochberg correlation [MS-MSB vs MS-MS] *p* < 0.05) but also the distance within BBG9-1-treated rats (Welch’s t-test with Benjamini-Hochberg correlation [MS-MSB vs MSB-MSB] *p* < 0.05) (Fig. 3b). The diversity of gut microbiota did not differ significantly between the control and MS groups, whereas it was lower in BBG9-1-treated rats than in controls at postnatal day 20 (F(2) = 4.47454, *p* < 0.05, Tukey-Kramer post hoc [Ctrl vs MSB] *p* < 0.05) (Fig. 3c).

At postnatal day 56, the structure of the gut microbiota was re-analyzed (Fig. 3a). When rats reached young adult age, we found no difference in structure of the gut microbiota among the three groups, suggesting that aging was likely to modify the gut microbiota structure acquired in the weanling period. Consistent with this finding, UniFrac distance analyses revealed no significant differences among the distances within normal rats, between control and MS rats, and between control and BBG9-1-treated rats (Welch’s t-test with Benjamini-Hochberg correlation *p* > 0.098). In addition, the distance between MS and BBG9-1-treated rats did not differ from the distance within MS rats ([MS-MSB vs MS-MS] *p* = 0.501) and the distance within BBG9-1-treated rats ([MS-MSB vs MSB-MSB] *p* = 0.452) (Fig. 3b). The diversity of gut microbiota showed no significant differences among the control, MS and BBG9-1-treated groups at postnatal day 56 (Kruskal-Wallis test *p* = 0.285) (Fig. 3c).

**Figure 4:**
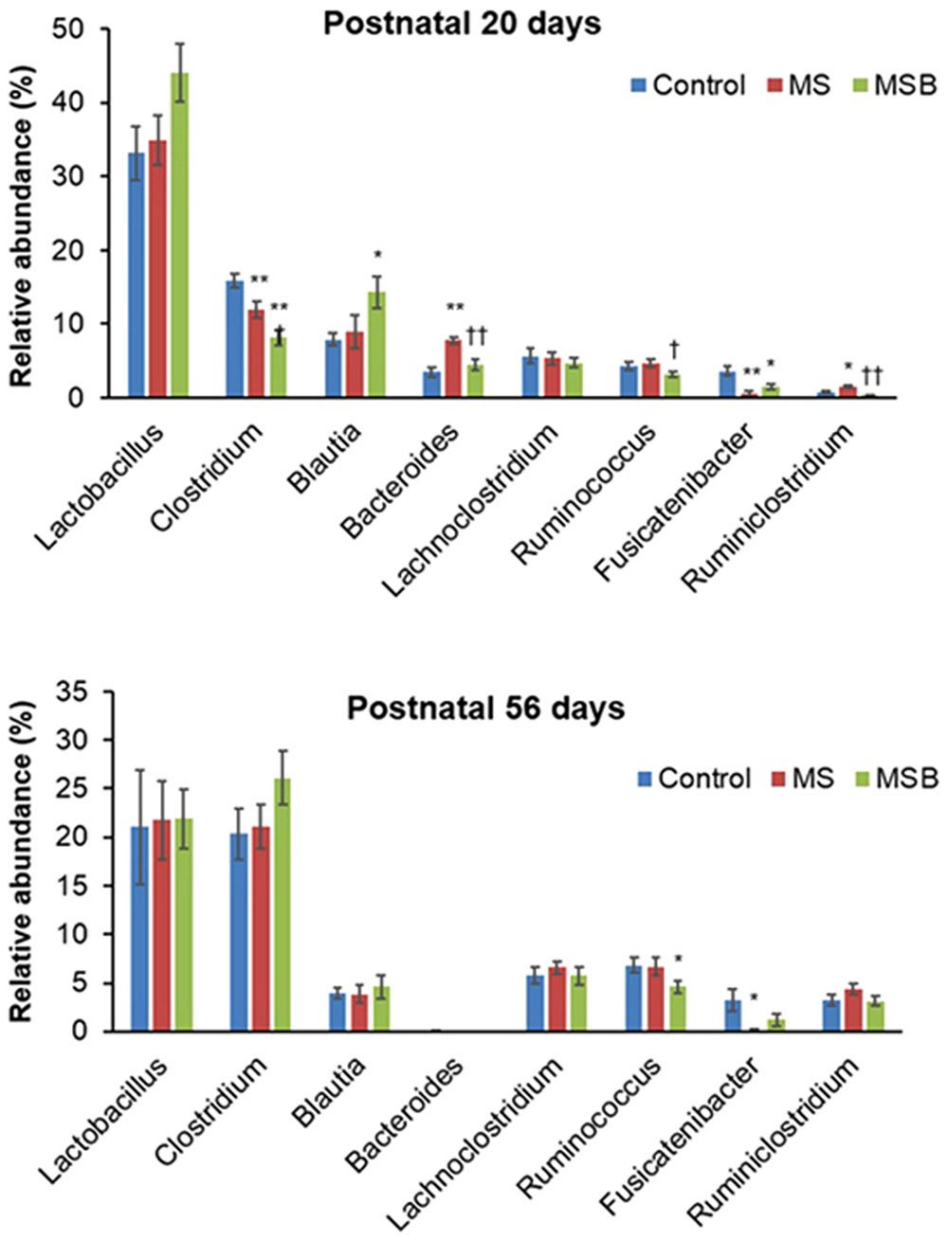
Effect of BBG9-1 on the relative abundance of intestinal bacteria in MS rats. The relative abundance of each bacterial genus was analyzed by next generation sequencing of bacterial 16S rDNA. MS, maternal separation; MSB, MS with BBG9-1 treatment (n = 10 in each group). Results are presented as the mean ± SE. *P < 0.05; **P < 0.01 vs control. ^†^*P* < 0.05; ^††^*P* < 0.01 vs MS group. Statistical significance was determined by Welch’s t test with Benjamini-Hochberg correlation.

Moreover, we examined the genera of gut microbiota present in the experimental rats. Among 8 major genera, *Bacteroides* was more abundant in MS rats than in control rats at postnatal day 20 (Welch’s t-test with Benjamini-Hochberg correlation [Ctrl vs MS] *p* < 0.01), while *Clostridium* and *Fusicatenibacter* were less abundant in MS rats than in control rats ([Ctrl vs MS] *p* < 0.05 and *p* < 0.01, respectively) (Fig. 4). The increase of *Bacteroides* was significantly reduced by the treatment with BBG9-1 ([MS vs MSB] *p* < 0.05). In MS rats, BBG9-1 treatment additionally reduced the abundance of *Clostridium* ([MS vs MSB] *p* < 0.05) but showed no effect on the abundance of *Fusicatenibacter*.

At postnatal day 56, *Fusicatenibacter* were still less abundant in MS rats than in control rats ([Ctrl vs MS] p < 0.05) but its abundance was not different between control and MSB groups ([Ctrl vs MSB] *p* = 0.133). *Ruminococcus* was less abundant in MSB than in MS group ([MS vs MSB] *p* < 0.05), being same at postnatal day 20. The other major genera showed no difference in abundance among the control, MS and MSB groups (Fig. 4).

### Effect of BBG9-1 on intestinal permeability in MS rats

**Figure 5:**
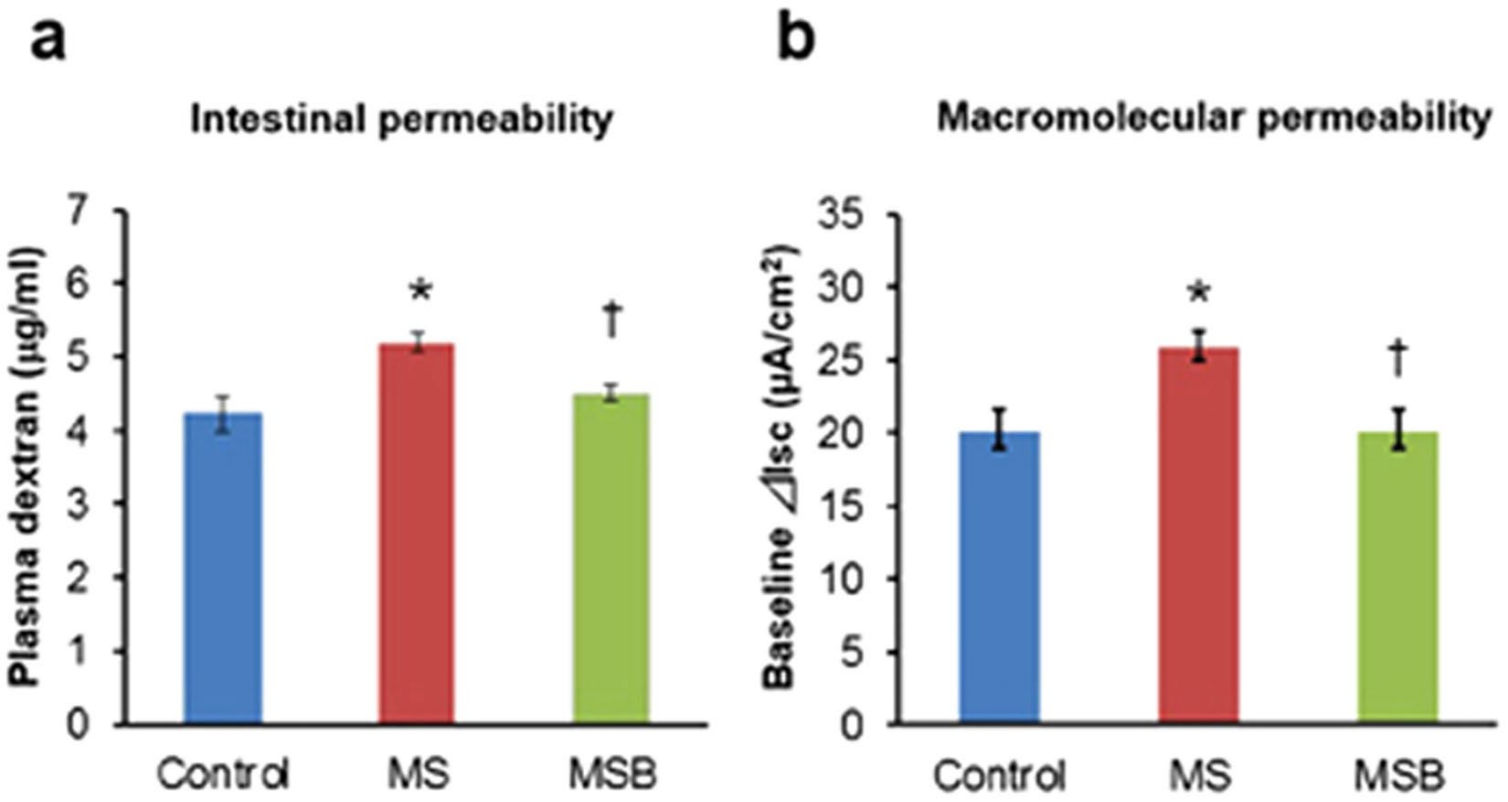
Effect of BBG9-1 on intestinal permeability in MS rats at postnatal day 20. (**a**) Intestinal permeability to FITC-labeled dextran (n = 6 in each group). (**b**) Macromolecular permeability assessed using the Ussing chamber system (n = 6 in each group). MS, maternal separation; MSB, MS with BBG9-1 treatment. Results are expressed as the mean ± SE. Significantly greater than control: *P < 0.05. Significantly smaller than the MS group: ^†^*P* < 0.05. Statistical significance was determined by Steel-Dwass test.

At postnatal day 20, the plasma level of FITC-dextran in MS rats was significantly higher than that in controls (F(2) = 8.410741, *p* < 0.01, Tukey-Kramer post hoc [Ctrl vs MS] *p* < 0.05) (Fig. 5a). BBG9-1 treatment restored the level of plasma FITC-dextran in MS rats to a level similar to that in the controls ([MS vs MSB] *p* < 0.05). The baseline Isc was significantly greater in the colon of MS rats than in that of controls (Kruskal-Wallis test *p* < 0.05, Steel-Dwass post hoc [Ctrl vs MS] *p* < 0.05) (Fig. 5b). Compatible with the data from the FITC-dextran assay, BBG9-1 treatment restored the level of the baseline Isc in MS rats to a level similar to that in the controls ([MS vs MSB] *p* < 0.05).

### Effect of BBG9-1 on HPA activity and defecation in MS rats subjected to restraint stress

**Figure 6:**
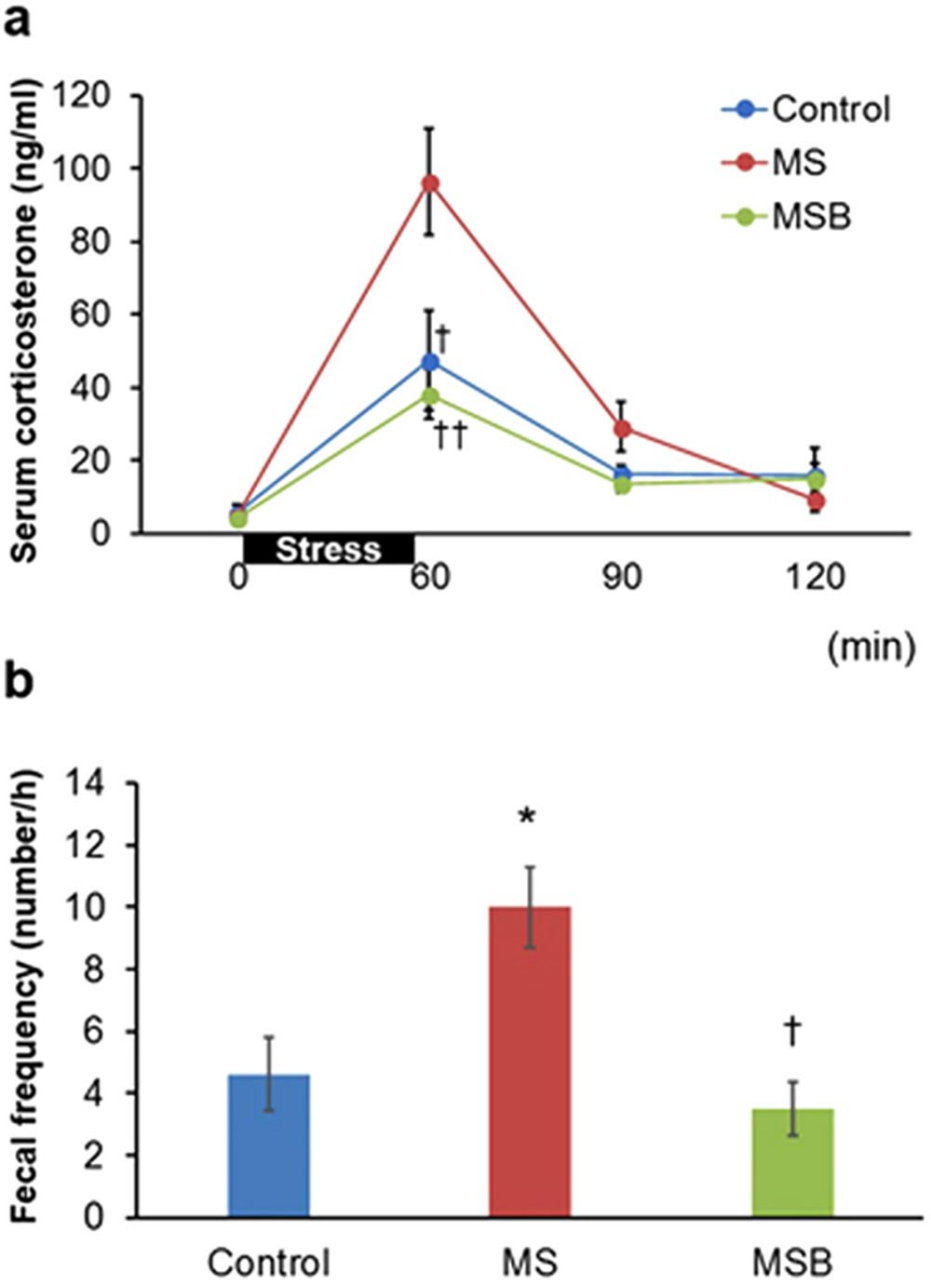
Effect of BBG9-1 on serum corticosterone level and fecal frequency in response to restraint stress in MS rats at postnatal day 56. (**a**) Time course of changes in the serum corticosterone level. Rats (n = 8 in each group) were subjected to a 1 h period of restraint stress (black bar). (**b**) Fecal frequency during restraint stress (n = 8 in each group). MS, maternal separation; MSB, MS with BBG9-1 treatment. Results are expressed as the mean ± SE. Significantly greater than control: *P < 0.05. Significantly smaller than the MS group: ^†^*P* < 0.05, ^††^*P* < 0.01. The significance of differences in corticosterone level and fecal frequency was determined by Tukey-Kramer and Steel-Dwass test, respectively.

When normal rats underwent restraint stress for 60 min, their serum corticosterone level began to increase, reaching a peak at the end of restraint, and then gradually falling again over a period of 120 min (Fig. 6a). A similar pattern was observed in not only the MS group but also the MSB group. However, the maximum level of serum corticosterone was significantly higher in the former than in the latter (F(2) = 6.609019, *p* < 0.01, Tukey-Kramer post hoc [Ctrl vs MS] *p* < 0.05, [MS vs MSB] *p* < 0.01), whereas no difference was detected between the control group and the MSB group.

In response to the restraint stress, the number of defecation was significantly increased in MS rats relative to the controls (F(2) = 9.752856, *p* < 0.01, Tukey-Kramer post hoc [Ctrl vs MS] *p* < 0.05) (Fig. 6b). However, the increase in the MS group was attenuated by treatment with BBG9-1 ([MS vs MSB] *p* < 0.05).

## Discussion

Accumulating evidence has suggested that alteration of gut microbiota is associated with the development of metabolic, psychological and gastrointestinal functional disorders^26,27^. The mechanism by which gut microbiota affects pathophysiology in those disorders remains largely unknown; however, it is believed that gut microbiota and host factors have important interactions^26,27^. The MS rat model, in which rat pups are subjected to psychological stress, is an animal model for human IBS, and indeed early life stress is closely associated with the development of FGIDs^16,17^. In this context, we have demonstrated that MS rats have an increased basal corticosterone level, suggesting increased activation of the hypothalamus-pituitary-adrenal axis. Furthermore, we have clarified that rats subjected to MS show a change in their intestinal permeability, being compatible with findings in human IBS^28,29^. It is noteworthy that the structure of the gut microbiota differs between controls and MS rats, suggesting that psychological stress is a host factor that can possibly modify the gut microbiota profile. These pathogenic features observed in MS rats are well consistent with those seen in IBS patients, thus confirming MS rats to be a suitable animal model of human IBS.

When the gut microbiota profiles of rats at postnatal days 20 and 56 were compared, all three treatment groups showed the same patterns of bacterial abundance in terms of *Ruminococcus* and *Fusicatenibacter*. Interestingly, a Finnish group have reported that the abundance of *Ruminococcus* is positively correlated with the IBS symptom score^30^, and *in vitro* studies have suggested that *Ruminococcus* may be involved in the breakdown of mucin^31^. In this context, the present study demonstrated that BBG9-1 treatment reduced the abundance of *Ruminococcus*, suggesting that BBG9-1 may have a protective effect on mucosal integrity. In this context of IBS, little information is available for the recently identified genus *Fusicatenibacter*. Interestingly, however, it has been reported that *Fusicatenibacter* decreases in patients with active ulcerative colitis (UC) but increases in UC patients who are in remission^32^. Thus, it is tempting to speculate that *Fusicatenibacter* may be associated with an anti-inflammatory effect, although accumulation of further data is needed. In our present animal model, *Fusicatenibacter* was significantly decreased in MS rats and tended to recover in MSB rats. Therefore, if *Fusicatenibacter* is associated with an anti-inflammatory action, its reduction in MS rats might have a detrimental impact on mucosal integrity.

Our main purpose in the present study was to examine whether the probiotic *Bifidobacterium bifidum* G9-1 exerts a beneficial effect on intestinal permeability, psychological stress and gut microbiota in MS rats. It was noteworthy that the increases observed in both intestinal permeability and the serum corticosterone level in MS rats were prevented by the treatment with BBG9-1. Although it is difficult to explain the mechanism responsible for this preventive effect, it is interesting that the structure of the gut microbiota in MS rats was shifted to one resembling that in control rats by the BBG9-1 treatment. Thus, it is tempting to speculate that the preventive effect of BBG9-1 on intestinal permeability and psychological stress may be due, at least in part, to an alteration of the gut microbiota. Of note, Otsuka *et al*. have reported that *Bifidobacterium* treatment during the neonatal period reduces the population of *Bacteroides* in the colon of normal weaning rats^33^. With regard to the specific bacteria responsible for the pathophysiological features in MS rats, we analyzed gut microbiota at the genus level and found an increase of *Bacteroides* and a decrease of *Fusicatenibacter*. Importantly, these alterations tended to be reversed toward control conditions after treatment with BBG9-1. Although the bacteria responsible for IBS remain unclear, it is interesting that we found an increase of *Bacteroides*, as this has also been reported in the gut flora of IBS patients^34–36^.

Our findings are of potential value in that we continued to observe the rats in the long term after the MS procedure. When we investigated the gut microbiota profile at postnatal day 56, we found no difference between controls and MS rats. However, this may not be surprising because in animal treated with antibiotics and/or specific foods, the modified structure of the gut microbiota is likely to return toward a normal situation, at least to a certain degree, in the long term after treatment^37–39^. In the present study, we also found no differences in the gut microbiota structure or serum corticosterone level between the controls and MS rats. However, it was interesting that MS rats showed hypersensitivity to restraint stress in terms of the serum corticosterone level and fecal frequency. These findings suggest that the stress-associated hypersensitivity in the brain-gut axis still remains even after the gut microbiota structure has recovered. Conversely, it is possible that early events in the weanling period may play a pivotal role in the initiation of hypersensitivity to stress. Whether or not gut microbiota is involved in the initiation of stress hypersensitivity is therefore a relevant issue. In this context, we clarified here that BBG9-1 treatment prevented the MS-associated alteration of gut microbiota and that MS treatment in the weanling period might help to ameliorate hypersensitivity to restraint stress at the young adult stage. Moreover, Barouei *et al*. have shown that maternal probiotic treatment partially protects against stress-induced disturbance of the adult gut microbiota^40^. These findings suggest that the gut microbiota plays some roles in the initiation of stress hypersensitivity, and that correction of any imbalance of gut microbiota structure using probiotics may be useful for preventing the initiation of stress hypersensitivity in this animal model of IBS.

In summary, we have shown that MS rats have pathogenic features similar to those consistently observed in patients with IBS, such as increased intestinal permeability, hypersensitivity to stress, and an imbalanced gut microbiota structure. Our findings also suggest that MS in the weanling period has a pivotal role in the initiation of hypersensitivity to stress, reflecting the well documented association between childhood abuse and the development of FGIDs^16,17^. Here, we would like to emphasize that in this animal MS model the gut microbiota was unequivocally involved in the initiation of hypersensitivity to stress, since treatment with the probiotic BBG9-1 corrected the gut microbiota structure, thus preventing such hypersensitivity. However, in the strict sense, our data were obtained during the periods from the weanling to the young adult stage, and not from mature adults. Therefore, it still remains to be clarified whether BBG9-1 treatment sustains its preventive effect against stress hypersensitivity until mature adulthood. Moreover, behavioral assessment of MSB rats for any specific behavioral phenotype^41,42^ remains an avenue of further investigation. In addition, since the effects of probiotics in IBS patients are still controversial^6–8^, in this context it would be interesting to examine the link between gut microbiota alteration and IBS-associated pathophysiological features in a future clinical trial.

## Electronic supplementary material

Supplementary Figure 1

## Data Availability

All data generated or analyzed during this study are included in this published article.
