# Author Correction: Effect of probiotic *Bifidobacterium bifidum* G9-1 on the relationship between gut microbiota profile and stress sensitivity in maternally separated rats

**DOI:** 10.1038/s41598-021-00491-4

**Published:** 2021-10-28

**Authors:** Hirokazu Fukui, Tadayuki Oshima, Yoshiki Tanaka, Yosuke Oikawa, Yutaka Makizaki, Hiroshi Ohno, Toshihiko Tomita, Jiro Watari, Hiroto Miwa

**Affiliations:** 1 Division of Gastroenterology, Department of Internal Medicine, Hyogo College of Medicine, Nishinomiya, Japan; 2 R&D Center, Biofermin Pharmaceutical Co., Ltd, Kobe, Japan; 3 Medical Science Department, Biofermin Pharmaceutical Co., Ltd, Kobe, Japan

Correction to: *Scientific Reports* https://doi.org/10.1038/s41598-018-30943-3, published online 17 August 2018

The original version of this Article contained an error in the legend of Figure 4.

“*P < 0.05; **P < 0.01 vs control. ^†^*P* < 0.05; ^†^*P* < 0.01 vs MS group.”

now reads:

“*P < 0.05; **P < 0.01 vs control. ^†^*P* < 0.05; ^††^*P* < 0.01 vs MS group.”

The original Article has been corrected.

