# Supplementary material for: Effect of probiotic Bifidobacterium bifidum G9-1 on the relationship between gut microbiota profile and stress sensitivity in maternally separated rats
Source: Sci Rep. 2018 Aug 17;8:12384. doi: 10.1038/s41598-018-30943-3 (PMC6098190; doi:10.1038/s41598-018-30943-3)
Supplement: Supplementary file 1 — Supplementary Figure 1 [file 41598_2018_30943_MOESM1_ESM.pdf]

Supplementary figures

Effect of probiotic *Bifidobacterium bifidum* G9-1 on the relationship between gut microbiota profile and stress sensitivity in maternally separated rats

Hirokazu Fukui, Tadayuki Oshima, Yoshiki Tanaka, Yosuke Oikawa, Yutaka Makizaki, Hiroshi Ohno, Toshihiko Tomita, Jiro Watari, Hiroto Miwa.

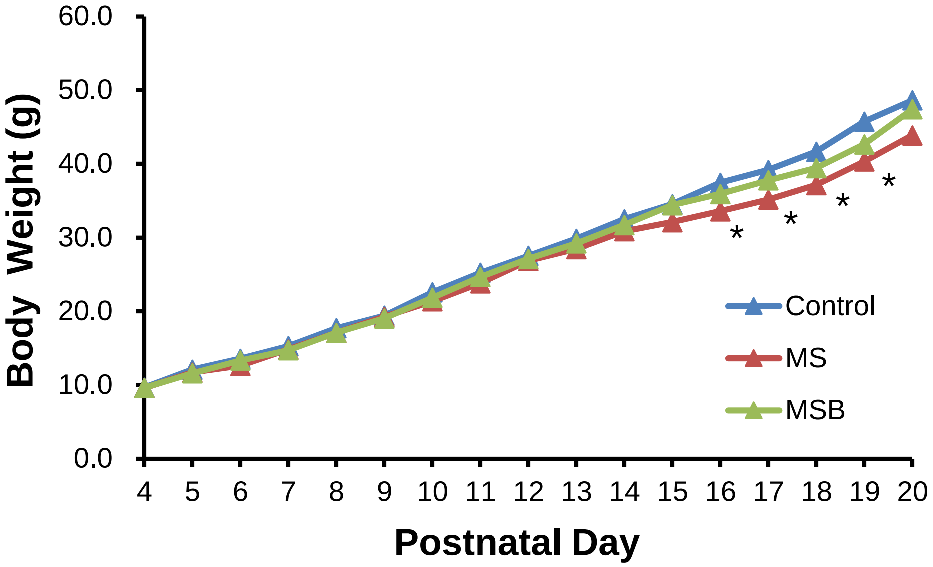

Supplementary Figure 1. Effect of BBG9-1 on body growth in female MS rats. Results are expressed as the mean ± SE (n = 6 female rats per group). Significantly smaller than in the control at the same time point: **P* <0.05 (Steel-Dwass test).
